# Author Correction: Rapid decline of neutralizing antibodies against SARS-CoV-2 among infected healthcare workers

**DOI:** 10.1038/s41467-021-23128-6

**Published:** 2021-05-10

**Authors:** Stéphane Marot, Isabelle Malet, Valentin Leducq, Karen Zafilaza, Delphine Sterlin, Delphine Planas, Adélie Gothland, Aude Jary, Karim Dorgham, Timothée Bruel, the Sorbonne Université SARS-CoV-2 Neutralizing Antibodies Study Group, Valérie Attali, Isabelle Baresse, Alexandra Beurton, Jacques Boddaert, Julie Bourmaleau, Martin Catala, Alexandre Demoule, Violaine Dunoyer, Cristina Esteban-Amarilla, Pierre Hausfater, Noémie Haziot, Queyras Ip, Nathalie Kubis, Laurence Lhoest, Catherine Lubetzki, Fabienne Marion, Elise Morawiec, Leila Mourtada, Brigitte Orcel, Capucine Morelot-Panzini, Mathieu Raux, Christophe Reinhard, Claire Riquier, Xavier Roubertier, Nicolas Weiss, Bernard Zalc, Sonia Burrel, David Boutolleau, Olivier Schwartz, Guy Gorochov, Vincent Calvez, Anne-Geneviève Marcelin

**Affiliations:** 1 Sorbonne Université, INSERM, Institut Pierre Louis d’Epidémiologie et de Santé Publique (iPLESP), Assistance Publique-Hôpitaux de Paris (AP-HP), Pitié Salpêtrière Hospital, Department of Virology, Paris, France; 2 Sorbonne Université, INSERM, Centre d’Immunologie et des Maladies Infectieuses (CIMI-Paris), AP-HP, Pitié Salpêtrière Hospital, Department of Immunology, Paris, France; 3 Virus and Immunity Unit, Department of Virology, Institut Pasteur, CNRS UMR 3569 Paris, France; 4 Vaccine Research Institute, Creteil, France; 5 Sorbonne Université, Assistance Publique-Hôpitaux de Paris, Pitié Salpêtrière Hospital, Paris, France

**Keywords:** Viral infection, SARS-CoV-2, Viral infection

Correction to: *Nature Communications* 10.1038/s41467-021-21111-9, published online 8 February 2021.

The original version of this Article contained errors in the figure legends for Figs. 1 and 2. Both of the legends incorrectly defined the whiskers of the box plots as representing “95% confidence intervals”. This has been corrected to “whiskers indicate the 1.5 interquartile range”.

This error has been corrected in both the PDF and HTML versions of the Article.

